# Box-and-arrow explanations need not be more abstract than neuroscientific mechanism descriptions

**DOI:** 10.3389/fpsyg.2014.00464

**Published:** 2014-05-22

**Authors:** Edoardo Datteri, Federico Laudisa

**Affiliations:** Department of Human Sciences, University of Milano-BicoccaMilano, Italy

**Keywords:** functional models, neuroscientific explanation, mechanisms, levels of analysis in neuroscience, regularities in neuroscience

## Abstract

The nature of the relationship between box-and-arrow (BA) explanations and neuroscientific mechanism descriptions (NMDs) is a key foundational issue for cognitive science. In this article we attempt to identify the nature of the constraints imposed by BA explanations on the formulation of NMDs. On the basis of a case study about motor control, we argue that BA explanations and NMDs both identify regularities that hold in the system, and that these regularities place constraints on the formulation of NMDs from BA analyses, and vice versa. The regularities identified in the two kinds of explanation play a crucial role in reasoning about the relationship between them, and in justifying the use of neuroscientific experimental techniques for the empirical testing of BA analyses of behavior. In addition, we make claims concerning the similarities and differences between BA analyses and NMDs. First, we argue that both types of explanation describe mechanisms. Second, we propose that they differ in terms of the theoretical vocabulary used to denote the entities and properties involved in the mechanism and engaging in regular, mutual interactions. On the contrary, the notion of abstractness, defined as omission of detail, does not help to distinguish BA analyses from NMDs: there is a sense in which BA analyses are more detailed than NMDs. In relation to this, we also focus on the nature of the extra detail included in NMDs and missing from BA analyses, arguing that such detail does not always concern how the system works. Finally, we propose reasons for doubting that BA analyses, unlike NMDs, may be considered “mechanism sketches.” We have developed these views by critically analyzing recent claims in the philosophical literature regarding the foundations of cognitive science.

## INTRODUCTION

**FIGURE 1:**
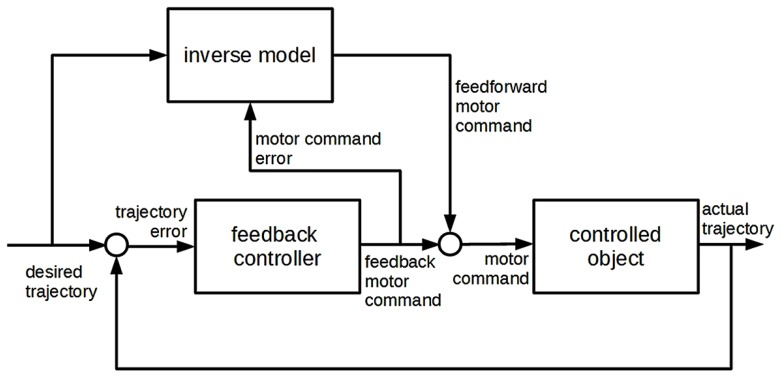
**A box-and-arrow model of motor control (adapted from Wolpert et al., 1998)**.

Explanations in the behavioral sciences take on a wide variety of styles. Quite often, especially at the early stages of their discovery, behavioral mechanisms are described without reference to brain areas or neural activity. The system is broken down into a number of interconnected components, each assumed to play an active part in the generation of the behavior to be explained. But no mention is made of what brain area, if any, corresponds to each component. For example, studies on motor control often postulate the existence of a “feedback controller” component in the system that produces motor commands on the basis of trajectory errors, without specifying which part of the target nervous system is presumed to perform this activity. When system components are only characterized on the basis of the activity they perform in the generation of the behavior to be explained, this is often referred to as a “box-and-arrow” (BA from now on) analysis of the system. An example of a BA analysis of motor control is shown in **Figure 1**. However, other behavioral mechanisms are described in terms of anatomically identified brain areas and the associated neural activities. For example, visually guided motor control in humans is thought to involve areas such as the visual cortex, the brain stem, the cerebellum and others. Similarly to BA explanations^1^, such neuroscientific mechanism descriptions (from now on NMDs) are often represented in box-and-arrow format in scientific papers (see **Figure 3** for an example); in contrast with what we have termed BA analyses, however, each box stands for a particular brain area or portion of the nervous system. Some brain areas may be linked to an activity performed within the framework of the behavior to be explained (e.g., in navigation when the hippocampus is said to hold a representation of space), but this is not always the case: a part of the nervous system may feature in an NMD even when the precise activity it carries out within the framework of the target behavior is not made explicit.

The theoretical vocabulary used in these two kinds of models is different, at least *prima facie*^2^. NMDs identify components and their organization using the language of neuroscience, which includes terms denoting brain areas, and expressions such as “neural activity,” “inhibitory connection,” “firing rate” and so on. In contrast BA explanations identify components and their organization in terms of a representational or information-processing language. For example, “feedback controllers” are said to produce internal representations of motor commands on the basis of a representation of trajectory error produced by other components. Yet most neuroscientists and philosophers of neuroscience assume that BA analyses and NMDs may be related to, and place constraints on, each other in some cases. Often, explanation of a behavior starts from the formulation of a BA analysis of the target system. Later, this BA analysis is taken as a basis on which to formulate an NMD by seeking out neural components that perform the activities specified in the BA analysis. In other cases, one starts with a NMD featuring interconnected brain areas, going on to identify the specific activities carried out by each: the NMD, in these cases, is taken as a basis on which to formulate a BA model of the system.

Now, it is one thing to assume that some kind of relationship holds between the two types of explanation, but quite another to clarify the nature of this relationship. What kind of constraints do BA analyses place on the formulation of NMDs, and vice versa? This is a question of great importance for neuroscientific research. Understanding the nature of these constraints would provide criteria for deriving NMDs from BA analyses in a principled way, and for testing the latter according to the empirical methods of the neurosciences. It would also contribute significantly to unifying branches of behavioral science, such as cognitive psychology (which typically adopts forms of BA analysis) and basic neuroscience, whose theoretical vocabulary may appear *prima facie* unrelated to each other. The aim of the present article is to take some further steps towards the formulation of such criteria on the basis of a close analysis of a case study on motor control in humans, and by critical examination of views recently expressed in the philosophical literature on the foundations of the cognitive sciences (Piccinini and Craver, 2011; Levy and Bechtel, 2013).

In the selected case study, a BA analysis of motor control was formulated, whose functional structure was claimed to correspond to the structure of a particular mechanism description couched in the vocabulary of neuroscience (Wolpert et al., 1998). The BA explanation and the NMD are described in Sections “On the Structure of Box-and-Arrow Models in Neuroscience” and “On the Structure Of Neuroscientific Mechanism Descriptions,” respectively. In Section “The Relationship Between Functional Models and Neuroscientific Mechanism Descriptions” we argue that, in both cases, a number of *regularities* were claimed to hold in the system, although different theoretical vocabulary was used to denote the entities and properties involved in these regularities. We further argue that these regularities played a crucial role in justifying the correspondence between the two explanations. Indeed, the formulation of an NMD proceeded by searching for neural groups whose activities conformed to the relationships expressed in the BA analysis. Thus, the correspondence between the two explanations seemed to consist, in the authors’ view, of a correspondence between the regularities expressed in each, while the BA analysis placed constraints on neuroscientific research given that it postulated a number of regularities to be sought out in the neural activity of the system.

In Section “The Relationship Between Functional Models and Neuroscientific Mechanism Descriptions,” we also take the selected case study to support a number of claims about the structural similarities and differences between BA analyses and NMDs. As far as the similarities are concerned we argue, consistently with what has been claimed, amongst others, by Piccinini and Craver (2011) that both explanations describe *mechanisms*, given that they refer to system components that interact with each other in a regular fashion^3^. In relation to the structural differences, we examine the claim that BA analyses are *less detailed* (Piccinini and Craver, 2011) or *more abstract* (Levy and Bechtel, 2013) than NMDs. We suggest that BA analyses may provide details that are missing from NMDs, namely, details on the representational roles played by certain neural groups, and that for this reason they may be said to be *richer*, or more detailed than NMDs. For the same reasons we also propose that the notion of *abstractness*, defined as “omission of detail” (Levy and Bechtel, 2013), does not help to define the difference between BA explanations and NMDs. Rather, the two kinds of explanation differ in relation to the theoretical vocabulary they use to denote system components. By changing theoretical vocabulary, and re-defining BA components in neuroscientific terms, one does not add crucial details about how the mechanism works: one simply describes the same boxes with different vocabulary. A way to add details on how the system works is, rather, to iterate mechanistic analysis on the components of a previously formulated system. This process, often referred to as *decomposition* in the epistemological literature on the cognitive sciences (Bechtel and Richardson, 1993), is to be viewed as distinct from the process of changing the theoretical vocabulary used to describe a mechanism.

We then examine more closely the claim, made by Piccinini and Craver (2011), that BA analyses are elliptical or incomplete versions of neuroscientific mechanism descriptions – *mechanism sketches*, in these authors’ terminology – insofar as they leave out crucial details on how the system works. We comment on this view by arguing that the details provided by NMDs and lacking in BA explanations need not concern how the system works. And we also suggest more general reasons for doubting that BA analyses may be considered elliptical versions of NMDs.

Let us begin this discussion by outlining the structure of the BA analysis of motor control proposed by Wolpert et al. (1998).

## ON THE STRUCTURE OF BOX-AND-ARROW MODELS IN NEUROSCIENCE

The scientific question addressed by Wolpert et al. (1998) is to understand how human beings control their movements along a desired trajectory – for example, how they successfully move a hand towards a specific object, or move an eye to track a portion of the visual environment. The idea proposed by the authors, and expressed in the BA analysis shown in **Figure 1**, is as follows.

A representation of the desired trajectory is available to the system. Then two components, the “feedback controller” and the “inverse model,” produce two motor commands – termed feedback and feedforward motor commands, respectively – that are combined before being sent, as a final motor command, to the “controlled object” (e.g., arm muscles) for execution. Both components produce motor commands, yet they there is a key difference between them. The “feedback controller” produces a motor command on the basis of “trajectory error,” i.e., on the basis of the difference between (1) the representation of the desired trajectory, and (2) the representation of the “actual trajectory” followed by the controlled object. This is the classical cybernetic negative-feedback principle (Rosenblueth et al., 1943), which is applied in many self-regulation devices (e.g., in thermostats). A major issue with such feedback-based control loops is the time required to receive feedback information on the actual trajectory. Sensory pathways are very delayed in humans, and a control mechanism based purely on feedback would make the system move too slowly or make too many errors. This was the main reason leading the authors to postulate a sort of shortcut, represented by the “inverse model” module. The function of this module is to generate motor commands on the basis of a representation of the desired trajectory only, with no sensory information available (intuitively, we use an inverse model when moving in our house in the dark). *Feedforward* motor commands are generated much more rapidly than the feedback ones, because they do not need to wait for the arrival and processing of sensory information. When a feedback command is available, it is combined with the feedforward command as earlier stated; otherwise, the system executes the feedforward command only, enabling itself to follow the desired trajectory within a reasonable time-frame.

Clearly, the “inverse model” must be trained before being able to generate the appropriate motor commands. The training signal consists of the representation of motor command error, generated on the basis of trajectory error (we correct our internal model of the house whenever we bump into a wall or piece of furniture that we erroneously believed to be farther away from us).

As mentioned in the Introduction, the authors of this study also formulated a neuroscientific mechanism description, discussed in detail in the next section, which was deliberately made to correspond with the structure described so far. As a basis for understanding the relationship between the two types of analysis, it is worth discussing some aspects of the BA explanation as described here. This explanation implies that there is something in the system which can *represent* desired trajectories. The system can also represent *feedback* and *feedforward motor commands.* Indeed, as explained before, the final motor command is a combination of feedback and feedforward motor commands: a plausible interpretation of this claim would be that the system has internal representations of the two commands, which are then combined into a third representation (the final motor command) driving the effector organs. In addition, the BA analysis refers to a number of functional components, including the “inverse model” and the “feedback controller,” presumed to be involved in motor control. These components are parts of the target system that are assumed to fulfill distinct functions within motor control.

This raises the question of what differentiates each component from the others. What is an “inverse model?” The authors of the study suggested that an inverse model is a component that transforms the desired movement trajectory of the controlled object into the motor commands required to attain this movement goal. That is to say that, by claiming that the system has an “inverse model,” the authors claimed that there is something in the system that establishes a *regular relationship* between desired trajectories and feedforward motor commands. This regular relationship was not precisely defined in their theory, apart from the claim that each desired trajectory is mapped onto the motor command *that would make the system follow that trajectory*^4^. Similarly, in suggesting that the system has a “feedback controller,” the authors claimed that there is something in the system that establishes a regularity between trajectory errors (which, in turn, depend on the difference between desired and actual trajectories) and feedback motor commands: feedback controllers produce motor commands that have the effect of reducing trajectory error^5^. These definitions are rather vague, but they nevertheless impose restrictions on the set of possible regularities associated with the “inverse model” and “feedback controller” components. The other functional components are associated with other regularities. To sum up, this BA model suggests that the system possesses a number of *internal representations* among which certain *regularities* hold.

**FIGURE 2:**
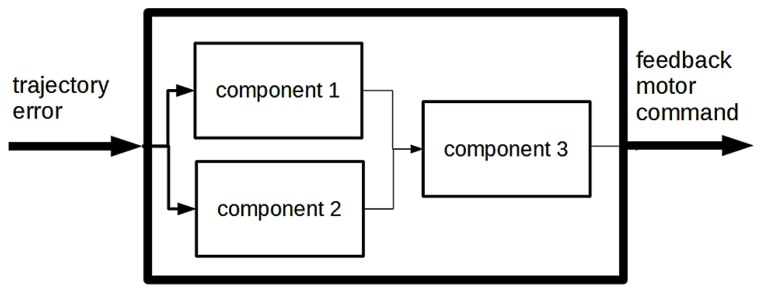
**A model of the “feedback controller” component of the BA model**.

Note that the BA analysis makes no claim about how each component establishes the corresponding regularity. As often noted in the philosophy of cognitive science, box-and-arrow analysis may be iterated to obtain finer-grained, more detailed BA analyses of the same behavior. See for example **Figure 2**, in which a purely notional BA subanalysis of the “feedback controller” component (not included in Wolpert et al., 1998) is shown. Three components are added, each of which establishes a regularity among additional intermediate representations. The relationship between the BA analysis described above, which we may call M for short, and the richer analysis M^′^, in which one or more functional components of M are further analyzed and broken down into a box-and-arrow structure, is often defined through appeal to the notion of *decomposition* (Rosenblueth and Wiener, 1945; Cummins, 1985; Bechtel and Richardson, 1993). Clearly, M^′^ may be further decomposed via an even finer-grained analysis; this process leads to the formulation of a *decomposition hierarchy* of BA explanations.

It is worth stressing here two aspects of BA decomposition that we come back to in the ensuing discussion. First, by decomposing a BA analysis, one obtains a richer BA analysis of the same system, in which further details are added on how the system is thought to work. For example, M is silent on a particular aspect of the functioning of the system, namely on how the “feedback controller” works, simply stating that the “feedback controller” component establishes a regular relationship between two representations. M^′^ adds details on how this component works, thus adding information on the functioning of the target system. Second, decomposition does not imply a change in the theoretical vocabulary used to describe the organization of the system – for example, specifically with regard to living systems, it does not imply a shift to the vocabulary of neuroscience – or vice versa. This is particularly evident in BA explanations formulated in computer science, in which components establishing regularities between system representations (typically expressed as functions in a given programming language) are analyzed into additional components (sub-functions) that establish regularities connecting additional system representations. Similarly, BA components in the study of cognition are often analyzed (decomposed) by postulating cascades of transformations among intermediate representations. Such a decomposition process does not lead to a change in vocabulary: it simply leads to another, richer, BA explanation. The process of decomposing a BA explanation must be kept conceptually distinct from the process of shifting to another theoretical vocabulary. Later in the paper, we focus on this distinction, arguing that the “translation” of a BA explanation into a neuroscientific MD does not necessarily lead to the addition of crucial details on the working of the system.

Furthermore, this distinction enables us to separate two methodological issues, both related to the more general problem of understanding the relationships between functional and mechanistic models in neuroscience. One of these issues is how to characterize the decomposition relationship, that is to say, the relationship holding between an explanation M and another explanation M^′^ obtained by decomposing from M and expressed using the same theoretical vocabulary. In other words, the issue of identifying the criteria used by scientists to transform previous explanations of a system into richer ones adding crucial details on the working of the system. A different issue is that of characterizing the relationship holding between two explanations of the same behavior formulated using different theoretical vocabularies. The case study analyzed here, as discussed in the next section, provides insights that help to address both questions, although this article is more strongly focused on the second issue.

## ON THE STRUCTURE OF NEUROSCIENTIFIC MECHANISM DESCRIPTIONS

**FIGURE 3:**
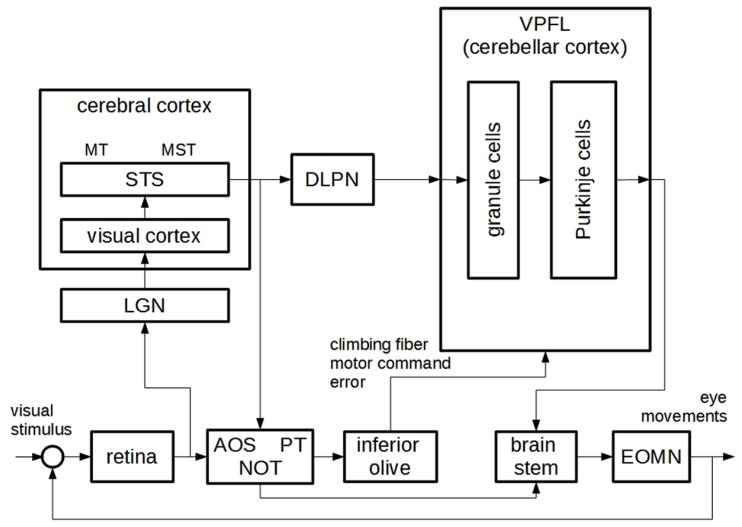
**A neuroscientific mechanism description for ocular-following reflex behavior, adapted from (Wolpert et al., 1998).** AOS: accessory optic system; PT: pretectum; NOT: nucleus of optic tract; EOMN: extra-ocular motor neurons; LGN: lateral geniculate nucleus; STS: superior temporal sulcus; MT: middle temporal area; MST: medial superior temporal area; DLPN: dorsolateral pontine nucleus; VPFL: ventral paraflocculus.

**Figure 3** shows a diagram formulated to explain how we^6^ control our eye movements to track moving portions of a visual scene (the so-called ocular following response or OFR). This motor control function cannot be fulfilled by feedback control only, given that visual feedback in humans is too delayed to enable efficient control of eye movements. The combination of feedback and inverse control, according to the principle described in the previous section, is a more promising approach to explaining this ability. A neuroscientific explanation of OFR was formulated by Wolpert et al. (1998) so as to correspond to the BA diagram represented in **Figure 1**. In particular, the authors claimed that the “inverse model” component corresponded to the component labeled as the ventral paraflocculus of the cerebellar cortex (VPFL) and to a number of additional components, as discussed below. The “feedback controller” corresponded to a set of components including the retina, the lateral geniculate nucleus (LGN), and portions of the visual cortex. Other components mentioned in their mechanistic analysis, as later discussed, cannot be easily mapped onto the BA explanation outlined in the previous section.

A question of primary importance is how the authors justified the claim that the two explanations corresponded to each other, albeit partially. Let us address this question by focusing on the “inverse model” component. The authors claimed that “the VPFL is the major site of the inverse dynamics model of the eye for OFR” (p. 341). This amounted to claiming that the VPFL is responsible for the fact that the activity of a neural group, which we call B for the moment, depends on the activity of another neural group A in the following regular fashion: the activity of B drives eye movements along the trajectory represented in A. In other words, by claiming that the VPFL served as an inverse model for the eye, Wolpert et al. (1998) were suggesting that the VPFL was responsible for a regular connection *of the “inverse model” type* between the activity of two neural groups. Should no neural activity in the brain be dependent in this way on a neural representation of desired trajectories, no inverse model would be claimed to be in the brain. The authors justified the claim that “the VPFL is the major site of the inverse dynamics model of the eye for OFR” by providing neuroscientific evidence for such a regularity. Note that an engineer would argue in the same way that an electromechanical electrical circuit included an inverse model, that is to say, by showing that the electrical activity at some point of a circuit depended on the electrical activity at another point of the circuit in accordance with “inverse model” regularity. In both cases, the fact that the BA analysis specifies *regularities* holding between parts of the system is crucial to understanding the nature of the relationship between BA and neuroscientific (or electromechanical) explanations of the same behavior.

The authors presented some interesting, albeit far from decisive, empirical support for their claim. First of all, the activity of certain VPFL cells – the Purkinje cells, often considered the output of the cerebellum – has been found to be regularly connected with eye movements. In particular, it is known that the activity of the Purkinje cells displays two types of spike, namely simple and complex spikes (SS and CS from now on; see Kandel et al., 2000). The SS are single action potentials. They occur at a relatively high frequency and have been found to correlate with certain aspects of eye movement. According to Wolpert et al. (1998), they may drive eye movements without waiting for the low-frequency arrival of sensory information, thus serving as feedfoward motor commands. Motor correlation has not been found in other neurons projecting from vision-related areas to the cerebellum, namely in the neurons of the dorsolateral pontine nucleus (DLPN) and the medial superior temporal (MST) area. In the authors’ view, given the absence of motor correlation and the connections with visual areas, these cells may “provide the desired trajectory information” to the cerebellum. Let us turn now to cerebellar CS. These are large-amplitude spikes followed by bursts of smaller action potentials. In addition they occur at a very low frequency (about 1–3 per second) and, similarly to the SS, display high correlation with eye movements. The authors suggested that the occurrence of a CS may signal the moment in which a motor command derived from feedback analysis (thus highly delayed, consistently with the low frequency of CS) interferes with the activity of the Purkinje cells and trains the inverse model. This relationship is shown in the BA diagram by the arrow connecting feedback motor commands with the inverse model. These empirical findings were taken by the authors as a basis for conjecturing that “the VPFL is the major site of the inverse dynamics model of the eye for OFR” (p. 341).

At the time of publication of Wolpert et al. (1998), the formulation of an NMD for OFR behavior was still at the early stages of development. However, on the basis of Wolpert et al.’s (1998) report, it is reasonable to believe that they were trying to identify a “neuroscientific version” of the regularities postulated by the BA analysis in the neural activity of the system. These regularities played a crucial role in justifying the correspondence between the BA analysis and the NMD: the VPFL, for example, was conjectured to be the neural site of the “inverse model” component as it supposedly establishes a regularity of the inverse-model type among entities and properties denoted with neuroscientific vocabulary, that is to say, between the neural activity of two neural groups. The justification was sought in the fact that the same regularities, expressed in different theoretical vocabularies, are found in the system. If no reference were made to the regularities postulated by the BA explanation – or if these regularities were specified in a qualitative and imprecise way, or BA components were only described in terms of textual expressions such as “feedforward controller” – it would not be clear how to relate the BA explanation to a neuroscientific mechanism description of the same system.

Note that, according to the regulative principle proposed here, a neuroscientific mechanism description may be formulated from a BA analysis by mapping each functional regularity onto a neural regularity, without adding components. This is the case of neuroscientific mechanism descriptions which reproduce the boxes and arrows of a BA analysis of the same system, while *adding* an indication of the neural structure subserving each functional role. In many cases, however, the shift from a BA explanation to a neuroscientific one is accompanied by a proliferation of neural structures.

This is also the case of the mechanism description analyzed here. Some neural structures internal to the VPFL are represented in the diagram. And various areas, such as the inferior olive and the previously mentioned AOS, PT, and NOT, are not easily mapped onto the BA explanation. Such a proliferation can result from a *decomposition* process, analogous to the process described in the previous section. We have pointed out that BA components may be analyzed into other BA sub-components, organized so as to produce the corresponding regularity (see **Figure 2**). By decomposing explanation M one obtains a richer explanation M^′^, which includes additional regularities internal to each component expressed in the same theoretical vocabulary. Similarly, the components of a neuroscientific mechanism may be analyzed into subcomponents, expressed using the theoretical vocabulary of neuroscience, and organized so as to produce the corresponding regularity. For example, when analyzing the internal VPFL cerebellar circuitry, additional regularities defining sub-components of the cerebellum may be identified: the richer neuroscientific mechanism is obtained by decomposition of the initial model. A case of decomposition in the study described here concerns the inferior olive, the AOS, the PT and the NOT, which the authors believed to be crucially involved in transforming the representation of the desired trajectory from sensory to motor coordinates, thus contributing to the inverse model transformation. Decomposition adds detail on the inner working of model components – thus, it provides extra detail about how the system is supposed to work – and for this reason it often marks an advance in the study of the modeled system^7^. However, it is worth stressing that it is one thing to “translate” a BA analysis into a neuroscientific mechanism description, and another to decompose the latter in order to obtain a more detailed model; and that – as often acknowledged in the philosophical literature – both BA explanations and NMDs may be decomposed, although they are expressed using different theoretical vocabularies.

## THE RELATIONSHIP BETWEEN FUNCTIONAL MODELS AND NEUROSCIENTIFIC MECHANISM DESCRIPTIONS

Let us sum up the claims made so far. In the case study analyzed here, a BA explanation and a NMD were introduced, each outlining a number of components supposedly involved in motor control, and describing their regular interactions. Notably, the neuroscientific mechanism description was claimed to “correspond” to the BA explanation. How did the authors justify this claim? As suggested in the previous sections, a key role in providing such a justification was played by the *regularities* expressed by the two explanations^8^: the authors seemed to follow the regulative principle according to which a BA analysis and a neuroscientific mechanism description “correspond” to each other to the extent that they display the *same* regularities, even though they are expressed in terms of different theoretical vocabularies. Indeed, the authors’ search for the neural structures corresponding to each BA component consisted of a search for the regularities characterizing the component, as outlined in the BA analysis, in anatomically connected regions of the brain. What makes something an “inverse model” is the fact that it establishes a particular regularity internal to the system, and what makes something a neural structure serving as an “inverse model” is the fact that it establishes a regularity of the inverse-model type among the activity of different neural groups^9^.

This provides a tentative answer to the key issue addressed in this article: what kind of constraints do BA analyses place on the formulation of NMDs, and vice versa? The BA analysis imposes constraints on the formulation of the NMD by postulating a number of regularities to be sought for in the neural activities of the system. Vice versa, the NMD constrains the space of the possible BA analyses of the system by postulating a number of neural regularities. Suppose that the study of a particular aspect of motor control in animals starts from the formulation of an NMD – possibly via the detection of correlations among the firing of different neural groups. Suppose, in addition, that one of these correlations takes a “feedback controller” form – e.g., the firing of neural group B drives muscles so as to reduce firing of neural group A. In that situation it would be reasonable to suppose that the system has a representation of the motor error (the firing of group A) and is able to produce an appropriate motor command to reduce motor error – in simpler terms, that it has a feedback controller. Should the regularity be different (for example, should the firing of A increase over time instead of tending to 0), one would not suppose that the system had a negative feedback controller (it might be thought to have a positive feedback controller instead).

The selected case study also provides a useful basis for assessing the structural similarities and differences between BA analyses and NMDs. As far as the similarities are concerned, both explanations specify a set of regularities supposedly holding in the system, though expressed using different theoretical vocabularies. And for this reason, consistently with Piccinini and Craver (2011), they both describe *mechanisms*. Indeed, if we are willing to consider the structure represented in **Figure 3** as a mechanism description, why not view BA analyses in the same way? Both types of explanation list a number of components suggested to be responsible for the behavior to be explained, and – more crucially – both specify the regular interactions holding among system components via a number of generalizations. The main difference between the two lies in the theoretical vocabulary used, but it is not clear why the choice of a particular theoretical vocabulary should determine whether or not to define something as a “mechanism description.”

As already stated, one of the major differences between the two mechanisms concerns the theoretical vocabulary used. The expression “theoretical vocabulary” is used here to denote a set of terms used in a particular discipline, or in a particular area of research, to express scientific theories. Statements regarding the neural activity of particular areas of the nervous system, and the anatomical connections among brain areas, are couched in the theoretical vocabulary of the neurosciences (which includes terms such as “neuron,” “neural activity,” “cerebellum,” “brain,” and so on). These terms are not used in what we refer to here as BA explanations^10^. As often pointed out in the philosophical literature on cognitive science, the theoretical vocabulary of BA explanations distinctively includes the term “representation.” Indeed, many BA explanations – including the explanation considered here – assume that the target system has a number of representations. And the various functional components, as in the case discussed here, are typically defined by appeal to these representations. Saying that the system has a “feedback controller” component is to make a rather amorphous claim, unless that component is defined more precisely as a component establishing a regular relationship between different representations held by the system. The notion of representation plays a key role in defining the components of a BA analysis and, therefore, in defining a BA explanation.

Do BA analyses and NMDs (also) differ in that the former are *less detailed* or *more abstract* than the latter? Such a position has been taken, amongst others, by Piccinini and Craver (2011), who propose that “functional and mechanistic explanations are not distinct and autonomous from one another precisely because functional analysis, properly constrained, is a kind of mechanistic explanation – an *elliptical* mechanistic explanation” (284). These authors call such elliptical mechanistic explanations *mechanism sketches*; therefore, in their view, functional explanations are mechanism sketches. BA analysis is a type of functional analysis, they propose, because it identifies components on the basis of the functional role they play in the framework of the behavior to be explained^11^. Piccinini and Craver’s (2011) identification of BA analyses with mechanism sketches is consistent with their broader view that BA analyses impose constraints on the formulation of neuroscientific mechanism descriptions. We agree with this hypothesis, but not with the hypothesis that BA analyses are elliptical or incomplete mechanism descriptions, for the following reasons.

As already discussed, both functional and neuroscientific mechanism descriptions specify a number of regularities occurring in the system. They differ in terms of the vocabulary used to denote the entities and properties involved in these regularities. Does a change in theoretical vocabulary entail a gain in completeness? One might answer in the affirmative, in light of the fact that the BA analysis does not convey information regarding the brain areas and neural groups underlying the various representations held by the system. The BA explanation, for example, does not specify what neural groups fulfill the role of representing desired trajectories or feedforward motor commands. This may lead one to believe that the BA explanation is less detailed than the NMD. However, it is also true that BA explanations convey information which are absent in NMDs. Indeed, in principle, NMDs need not provide information on the representational functions of the neural groups involved in the mechanism. They may simply identify neural components and define their regular interaction, without claiming, for example, that the firing activity of neural group A is responsible for, encodes, or serves as, a representation of something. Functional information about the system’s representational abilities is explicitly provided by BA explanations but may be missing from NMDs^12^. Therefore, BA explanations may convey details that are lacking in NMDs. For these reasons, one may legitimately view NMDs as “more detailed” than BA explanations only by appropriately restricting the term “detail” to refer to “*neural* detail.” But the awarding of such an epistemic privilege to neural details requires justification.

These considerations may also be applied to Levy and Bechtel’s (2013) views on *abstractness*, defined as “omission of detail.” BA explanations omit details provided by NMDs, and vice versa. For this reason, they cannot be ordered on a scale of abstractness without being explicit about the nature of the details at stake (BA explanations are more abstract than NMDs as far as neural details are concerned, and NMDs are more abstract than BA explanations as far as representational details are concerned)^13^. We claim that a better way to define the difference between the two kinds of explanation is to say that they each convey different information (with each abstracting with respect to details of a particular kind) about the target system, by using different theoretical vocabularies.

Let us further elaborate on the nature of the details omitted from BA explanations and provided by NMDs, by recalling that Piccinini and Craver (2011) describe BA analyses as mechanism sketches, which they discuss in the following terms.

Descriptions of mechanisms [...] can be more or less complete. Incomplete models – with gaps, question-marks, filler-terms, or hand-waving boxes and arrows – are mechanism sketches. Mechanism sketches are incomplete because they leave out crucial details about how the mechanism works. Sometimes a sketch provides just the right amount of explanatory information for a given context (classroom, courtroom, lab meeting, etc.). Furthermore, sketches are often useful guides to the future development of a mechanistic explanation. Yet there remains a sense in which mechanism sketches are incomplete or elliptical (p. 292).

Now, it is one thing to claim that BA explanations are elliptical with respect to neuroscientific mechanism descriptions given that they do not provide information on the neural areas subserving the various representational roles, but another to claim that they are elliptical because they “leave out crucial details about how the mechanism works.” By changing theoretical vocabulary, and expressing similar regularities in the language of neuroscience, one does not add crucial details about how the mechanism works: one simply describes the same boxes with a different vocabulary. Answers to questions such as “how does system A work?” take the form of mechanism descriptions; further detail on how system A works is added by decomposing the mechanism description, as illustrated in the previous sections, and not by expressing it with a different vocabulary^14^. If one knows that the target system has a component X, simply defining that component using a different vocabulary does not *enrich* the mechanism description of the system – rather it *translates* it into another description. This is not to deny that such a translation may mark important progress in the study of the target system, possibly because it paves the way for the application of additional experimental techniques. And it is true that sometimes – as in the present case study – the shift from a functional to a neuroscientific mechanism description is accompanied by a decomposition process. But this need not be always the case. The point to be emphasized is that changing theoretical vocabulary in the description of a system should be clearly distinguished from the process of decomposing a model of the system. This distinction, as already pointed out, has the effect of splitting the question of the relationship between different models of a system into two questions: the first concerns the relationship between models expressed using different vocabularies, while the second concerns the relationship between different levels of the decomposition hierarchy.

This view has another implication in relation to Piccinini and Craver’s (2011) claim that BA analyses are mechanism sketches, that is to say, incomplete or elliptical. Are NMDs mechanism sketches too? If so, the notion of mechanism sketch would not help to draw a distinction between BA analyses and NMDs, contrary to the main point made by Piccinini and Craver (2011). It follows that NMDs, for Piccinini and Craver, are not mechanism sketches. And the assumption that BA explanations, as mechanism sketches, are elliptical and incomplete, leads us to conclude that NMDs are not elliptical nor incomplete, namely, that they are *complete* descriptions of a mechanism. It is important to be careful and explicit about the sense in which NMDs may be defined as such, in order to avoid the strong implication that NMDs say *everything* – being complete descriptions – that can be said about a mechanism (e.g., to avoid the implication that the mechanism description relating to long-term potentiation used by Craver, 2002 to illustrate the notion of “mechanism description” says everything about the target mechanism). Here we have claimed that NMDs may omit information about the representational roles of the neural structures in the target system and, that, for this reason, they may sensibly be view as incomplete with respect to BA explanations.

## CONCLUSION

The nature of the relationship between box-and-arrow explanations, which do not invoke neural mechanisms, and neuroscientific mechanism descriptions, is a key foundational issue for cognitive science. On the one hand, the opportunity to disregard neural details in the explanation of behavior has in the past been a source of insight and creativity, yielding hypotheses that led to a better understanding of numerous behavioral and cognitive phenomena. On the other hand, the strong increase in detail of analysis, both theoretically and experimentally, on the part of the neurosciences has led to a corresponding increase in the production of models whose cognitive significance, however, is still far from clear and unequivocal. In the present article we have attempted to tackle the question from a foundational viewpoint, by focusing on the nature of the relationship between box-and-arrow, non-neural explanations of behavior, and neuroscientific mechanism descriptions. On the basis of a case study concerning motor control, we first argued that the regularities formulated in box-and-arrow explanations and neuroscientific mechanism descriptions play a crucial role in justifying any “correspondence” between the two. The regularities formulated in BA explanations place constraints on the formulation of NMDs, and vice versa. Then, we made some general claims regarding the similarities and differences between BA analyses and NMDs. As far as the similarities are concerned, consistently with other positions expressed in the literature, we argued that both kinds of explanations describe mechanisms. As far as the differences are concerned, we suggested that the two kinds of explanation differ in terms of the theoretical vocabulary used to denote the entities and properties involved in the mechanism and engaging in regular, mutual interaction. On the basis of the selected case study we also argued, first, that the notion of abstractness, defined as omission of detail, does not help to distinguish BA analyses from NMDs. BA analyses are more abstract than NMDs with respect to a particular class of detail, but may be less abstract with respect to another class of detail. Second, we argued that the details added into NMDs and missing from in BA explanations need not necessarily concern how the system works. Third, we have proposed reasons for doubting that BA analyses, unlike NMDs, may be considered mechanism sketches. These views are based on a critical examination of claims made by Piccinini and Craver (2011) and Levy and Bechtel (2013). Taken together, they may contribute to further clarifying the relationship between different styles of explanation widely adopted in behavioral sciences, and, therefore, to unifying branches of cognitive science that adopt markedly different theoretical vocabularies.

## Conflict of Interest Statement

The authors declare that the research was conducted in the absence of any commercial or financial relationships that could be construed as a potential conflict of interest.

## Footnotes

1 The expressions “BA analysis” and “BA explanation” will be used interchangeably in this article.

2 The intended meaning of the expression “theoretical vocabulary” will be clarified in Section “The Relationship Between Functional Models and Neuroscientific Mechanism Descriptions.”

3 For the purposes of the present paper, we provisionally accept the analysis of neuroscientific mechanisms provided by Craver (2007).

4 More precisely, the regularity associated with the “inverse model” module in the BA diagram links *three* representations to one other, as shown in **Figure 1**: the feedforward motor command depends on the desired trajectory and on the motor command error, which intermittently trains the motor apparatus’ internal model.

5 The “trajectory error” and the “feedback motor command” representations may well be regarded as the *input* and the *output* of the “feedback controller” component, respectively. However, the use of these terms, although consistent with the present analysis, would require an additional account of what makes something an “input” or an “output” of a BA component – an account which may enable one to understand, e.g., why the “trajectory error” is more properly regarded as an input rather than as an output of the “feedback controller” component. Such an account is not really required for the purposes of the present article. For this reason we provisionally avoid the use of the terms “input” and “output” and only claim that, according to the BA analysis, the “feedback controller” is a component that establishes a regular relationship between the two representations.

6 This study actually examined monkey OFR, and the neuroscientific mechanism description discussed below refers to areas of the monkey brain. However, the authors suggested that this mechanism description could also shed light on the OFR mechanism in humans. Discussion of the reasons for such a generalization is beyond the scope of the present article.

7 As discussed in Footnote 14, this is not to say that decomposition leads always to better explanations. We wish to remain entirely neutral in relation to what level of explanation, or which theoretical vocabulary, is more suited to the purpose of explaining adaptive behavior or answering specific why-questions about it. Our point solely concerns the structural relationship between BA explanations and NMDs.

8 Whether it is possible to have a theory of what we mean by a “regularity” in the field of neurosciences is, to a large extent, an open question. Woodward’s notion of “invariance under interventions” (Woodward, 2003, 2010) is a useful starting point to address this issue. Many authors – including Craver (2007) – have claimed that neuroscientific generalizations are *fragile* and exception-ridden, given that they are subject to a formidable number of boundary conditions. This claim may be taken to pose a serious problem for present analysis: if no robust (as opposed to fragile) generalization may be found in the neural structure of the system, how can the correspondence between the BA explanation and the NMD be justified according to the regulative principle proposed here? This is a legitimate question, needing to be addressed by further analysis; nevertheless, we believe that the regulative principle put forward here is reasonable. Indeed, in our opinion, it is a matter of fact that Wolpert et al. (1998) identified regularities in the neural system. These regularities were claimed to be robust enough to be found in different individuals and at different times. Clarifying how neuroscientific generalizations may be taken as sufficiently robust to license prediction and explanation, despite being subject to a multitude of boundary conditions, is in our opinion an important aim for the epistemological analysis of neuroscience; see the discussion in Datteri and Laudisa (2012).

9 Anatomical considerations guide the authors in selecting the regularities which may play a part in the explanation. Indeed, only the regularities holding among anatomically connected parts of the system are typically included in the description of an explanatory mechanism. This is consistent with the view proposed here: to claim that neuroscientific mechanism descriptions formulate regularities holding in the system does not mean to claim that *any* regularity may be included in a description of a mechanism.

10 According to the definition proposed in Section “Introduction,” BA explanations do not specify which parts of the target nervous system are presumed to perform the activities mentioned there. For this reason NMDs are not included in the class of BA explanations, even though (as noted before) NMDs are often represented in a box-and-arrow format in scientific papers. BA and NMDs, as defined here, differ in relation to the theoretical vocabulary used to denote system components. This is not to say that the use of representational terms is incompatible with the use of neuroscientific terms in the same explanation. Indeed, in the course of scientific discovery, researchers often formulate “mixed” explanations using *both* representational and neuroscientific terms. An example can be found in our case study (Wolpert et al., 1998). After describing the BA explanation, the authors propose an analysis of motor control (**Figure 1B** at p. 339) which provides information on the neural localization of the “inverse model” component *only*. This analysis, presented by the authors as an intermediate stage in the formulation of an NMD from the initial BA explanation, uses representational and neuroscientific terms. Nothing, in the view proposed here, rules out the possibility of formulating mixed models of that kind. Rather, the analysis proposed here – focused on the relationship holding between non-mixed BA explanations and NMDs – may also contribute to understanding how mixed analyses are formulated from explanations of the former or the latter kind. Indeed, there are reasons to believe that the mixed analysis in (Wolpert et al., 1998) is obtained from the BA explanation by applying the criteria discussed here to one component only, i.e., by searching for an “inverse-model” type regularity in the neural structure of the target system, and provisionally ignoring the other BA components.

11 We consider Piccinini and Craver’s (2011) views to be relevant to the main points made here, given that we believe that they would classify the analysis represented in **Figure 1** as a functional analysis. Indeed, each of its components is labeled using an expression that denotes a functional role, i.e., “feedback controller” and “inverse model.” And the notion of representation, used to characterize the various components, is functional by definition.

12 This is not to say that NMDs are not *functional*. Indeed many authors, including Piccinini and Craver (2011), have convincingly shown that NMDs are functional according to various interpretations of the term. Not least because they identify neural components that are assumed to play a crucial functional role in the framework of the behavior to be explained. But NMDs do not always convey the particular kind of functional information provided by BA explanations. Specifically, they may include reference to brain areas thought to play a crucial functional role in the mechanism (e.g., the cerebellar cortex) without defining their functional role (e.g., inverse control) or refer to the activity of a particular neural group without providing information on what this activity is thought to represent. As a matter of fact, neuroscientists often *add* this kind of functional detail to purely anatomical NMDs for a range of explanatory or experimental purposes.

13 Levy and Bechtel’s (2013) study concerns the relationship between NMDs and models that “abstract from the structural specifics of a mechanism and represent it in a skeletal, coarse-grained manner.” In these models, “the pattern of causal relations within a system is highlighted, while structural aspects of components are suppressed” (241). Such models may be sensibly viewed as more abstract than NMDs, because they are obtained from NMDs by omitting certain sorts of details. However, we doubt that many BA explanations in the cognitive sciences, including the analysis represented in **Figure 1**, may be classified as abstract models of that kind. The reason is that these explanations are obtained from NMDs by omitting neural details *and* by adding representational details: they do something more than representing the pattern of causal relations within the target system. Indeed, it is possible to formulate an abstract model both of the NMD shown in **Figure 3** *and* of the BA analysis shown in **Figure 1**. Suppose, for example, that a “feedback controller” is defined as a component that generates a motor command whose intensity, represented by a real number, is inversely proportional to the intensity of an error, represented by another number, as determined by coefficient *a*. Thus the abstract model of this BA component will take the form *y* = *ax*. This abstract model is not the same thing as the BA analysis on which it is modeled.

14 Adding details on how the system works is not the same as providing a better explanation of the target behavior: the added details could well be irrelevant for the given explanatory purpose, for reasons not explored here. We wish to maintain a neutral position with regard to what decomposition level is the most suitable for explaining behavior, and with regard to whether only NMDs can explain. Our sole interest is the relationship between BA analyses and NMDs. For this reason, we do not comment on the claims made by Levy and Bechtel (2013) and by Piccinini and Craver (2011) in relation to the explanatory power of BA analyses and NMDs.
